# Performance Evaluation of zk-SNARK Protocols for Privacy-Preserving Sensor Data Verification: A Systematic Benchmarking Study

**DOI:** 10.3390/s26082486

**Published:** 2026-04-17

**Authors:** Oleksandr Kuznetsov, Yelyzaveta Kuznetsova, Gulzat Ziyatbekova, Yuliia Kovalenko, Rostyslav Palahusynets

**Affiliations:** 1 Department of Theoretical and Applied Sciences (DISTA), eCampus University, Via Isimbardi 10, 22060 Novedrate, Italy; 2 Department of Intelligent Software Systems and Technologies, School of Computer Science and Artificial Intelligence, V. N. Karazin Kharkiv National University, 4 Svobody Sq., 61022 Kharkiv, Ukraine; 3 Department of Computer Science “Giovanni degli Antoni”, Università degli Studi di Milano, Via Festa del Perdono, 20122 Milan, Italy; yelyzaveta.kuznetsova@studenti.unimi.it; 4 Department of Computer Engineering, Almaty Technological University, 050012 Almaty, Kazakhstan; 5 Department of Artificial Intelligence and Big Data, Faculty of Information Technology, Al-Farabi Kazakh National University, 050040 Almaty, Kazakhstan; 6 Institute of Information and Computational Technologies of the Science Committee of the Ministry of Science and Higher Education of the Republic of Kazakhstan (SC MSHE RK), 050010 Almaty, Kazakhstan; 7 Department of Artificial Intelligence, State University of Information and Communication Technologies, 7, Solomyanska Str., 03110 Kyiv, Ukraine; kovalenko@umsf.edu.ua; 8 Permanent Mission of Ukraine to the International Organizations in Vienna, 1010 Vienna, Austria

**Keywords:** zero-knowledge proofs, zk-SNARK, Groth16, sensor networks, privacy-preserving verification, performance benchmarking, IoT security, blockchain

## Abstract

The proliferation of sensor networks in critical infrastructure, healthcare monitoring, and smart city applications demands robust privacy-preserving mechanisms for data verification. Zero-knowledge succinct non-interactive arguments of knowledge (zk-SNARKs) offer a promising cryptographic primitive that enables data integrity verification without revealing sensitive sensor readings. However, the practical feasibility of deploying zk-SNARKs in resource-constrained sensor network environments remains insufficiently characterized. This paper presents a systematic benchmarking study of the Groth16 zk-SNARK protocol across eight representative circuit types spanning six orders of magnitude in computational complexity, from basic arithmetic operations (1 constraint) to ECDSA signature verification (1,510,185 constraints). Using an automated open-source benchmarking framework built on the Circom-snarkjs toolchain, we conducted 160 statistically controlled measurements (20 iterations per circuit) with cold/warm separation, collecting proof generation time, verification time, proof size, memory consumption, and witness generation overhead. Our results demonstrate that Groth16 proofs maintain a constant size of 804.7±1.7 bytes and near-constant verification time of 0.662±0.032 s regardless of circuit complexity, with coefficients of variation below 5% across all circuit types. Proof generation time exhibits sub-linear scaling (α=0.256, $R^{2}=0.608$), with statistically significant differences between circuit categories confirmed by one-way ANOVA (F=355.0, $p<10^{-79}$, $\eta^{2}=0.94$). We identify three operational deployment tiers for sensor network architectures and estimate energy budgets for battery-powered devices. These findings provide actionable guidance for the design of privacy-preserving data verification systems in next-generation sensor networks.

## 1. Introduction

The rapid expansion of sensor networks across industrial monitoring, healthcare systems, environmental sensing, and smart city infrastructure has created unprecedented volumes of data that require reliable integrity verification [1]. Traditional approaches to sensor data authentication rely on centralized trust authorities and symmetric key management, which introduce single points of failure and scalability limitations [2]. As sensor networks increasingly underpin critical decision-making in areas such as patient health monitoring and environmental hazard detection, the consequences of data manipulation or unauthorized access become severe.

Blockchain technology has emerged as a promising paradigm for decentralized sensor data management [3], providing tamper-proof audit trails and transparent verification mechanisms [4]. In vehicular and IoT environments, blockchain-assisted authentication schemes have demonstrated the potential of decentralized trust architectures, including conditional privacy-preserving batch verification [5] and anonymous authentication with adaptive group key agreement [6]. However, recording raw sensor data on public or consortium blockchains raises significant privacy concerns, particularly in healthcare and industrial settings where sensor readings may constitute sensitive personal or proprietary information [7]. This tension between transparency and privacy represents a fundamental challenge for blockchain-enabled sensor networks.

Zero-knowledge proofs (ZKPs) offer an elegant resolution to this tension by enabling one party (the prover) to convince another party (the verifier) that a computational statement is true without revealing any information beyond the validity of the statement itself [8]. Among ZKP constructions, zero-knowledge succinct non-interactive arguments of knowledge (zk-SNARKs) [9] are particularly attractive for sensor network applications due to three key properties: (1) *succinctness*—proofs are extremely compact regardless of computation complexity; (2) *non-interactivity*—proofs can be verified without real-time communication with the prover; and (3) *efficiency*—verification is computationally inexpensive compared to re-executing the original computation.

These properties align well with the constraints of sensor network environments, where communication bandwidth is limited, real-time interaction may be infeasible, and verification must be performed on resource-constrained devices. Practical applications include privacy-preserving sensor data attestation (proving that a temperature reading falls within a safe range without revealing the exact value), anonymous device authentication (proving possession of valid credentials without disclosing device identity), and verifiable data aggregation (proving correct computation of aggregate statistics without exposing individual readings) [10].

Despite growing theoretical interest, the practical performance characteristics of zk-SNARK implementations in contexts relevant to sensor networks remain insufficiently characterized. Existing benchmarking studies tend to focus on specific applications [11] or theoretical complexity analysis [12] without providing comprehensive empirical data across the spectrum of circuit complexities encountered in sensor network scenarios. Critical questions remain unanswered: What are realistic proof generation and verification times for circuits representative of sensor data verification tasks? How do proof sizes compare with typical sensor network communication constraints? At what complexity level does proof generation become impractical for various classes of sensor network devices?

This paper addresses these gaps through a systematic empirical evaluation of the Groth16 zk-SNARK protocol—the most widely deployed zk-SNARK construction—across eight carefully selected circuit types that represent common computational patterns in sensor network applications. Our circuits span six orders of magnitude in constraint count (from 1 to over 1.5 million), covering basic data validation, range proofs for threshold checking, cryptographic hash verification for data integrity, Merkle tree membership proofs for set verification, and digital signature verification for authentication.

The main contributions of this work are as follows:

1. We present the first systematic benchmarking study specifically designed to evaluate zk-SNARK feasibility for sensor network data verification, covering eight representative circuit types across six orders of magnitude in computational complexity.
2. We provide detailed empirical measurements of all phases of the Groth16 proof lifecycle (compilation, setup, witness generation, proof generation, and verification), enabling realistic resource planning for sensor network deployments.
3. We identify and characterize three operational deployment tiers based on circuit complexity and resource requirements, providing actionable guidelines for mapping sensor network verification tasks to appropriate computational infrastructure.
4. We release an open-source automated benchmarking framework that enables reproducible performance evaluation and facilitates future comparative studies across different zk-SNARK implementations and hardware platforms.
5. We propose a five-layer reference architecture for privacy-preserving sensor data verification with blockchain anchoring, demonstrating how the empirical benchmarking results translate into a deployable IoT system design with GDPR-compliant data processing and immutable audit trails.

The remainder of this paper is organized as follows. Section 2 reviews related work on ZKP applications in IoT and sensor networks. Section 3 provides the necessary cryptographic background. Section 4 describes our experimental methodology and benchmarking framework. Section 5 presents the experimental results. Section 6 discusses implications for sensor network deployment and identifies operational tiers. Section 7 concludes with a summary and future research directions.

## 2. Related Work

Research at the intersection of zero-knowledge proofs and sensor networks has gained significant momentum in recent years, driven by the increasing demand for privacy-preserving data verification in IoT ecosystems.

### 2.1. Zero-Knowledge Proofs in IoT and Sensor Networks

Several studies have explored the application of ZKP techniques to IoT environments. Baza et al. [13] proposed a blockchain-based authentication scheme for IoT devices using zk-SNARKs to protect device identity during network access. Their work demonstrated the feasibility of ZKP-based authentication but did not provide comprehensive performance characterization across different circuit complexities. Bai et al. [14] investigated lightweight ZKP protocols for resource-constrained devices, focusing on reducing computational overhead through optimized circuit design. While their theoretical analysis showed promising complexity reduction, empirical validation was limited to a single circuit type.

Steffen et al. [15] examined ZKP-based data integrity verification for industrial sensor networks, proposing a framework that uses Merkle proofs combined with zk-SNARKs for privacy-preserving audit trails. Their results highlighted the importance of proof size constraints for low-bandwidth industrial communication protocols but did not systematically evaluate the relationship between circuit complexity and resource consumption.

### 2.2. zk-SNARK Performance Evaluation

The performance characteristics of zk-SNARK systems have been studied from various perspectives. Ben-Sasson et al. [16] provided foundational theoretical complexity analysis for zk-SNARK constructions, establishing the O(mlogm) computational complexity bound for proof generation, where *m* denotes the number of constraints in the arithmetic circuit. Subsequent empirical studies have sought to validate these theoretical predictions.

Ozdemir and Boneh [17] conducted a comparative evaluation of multiple zk-SNARK backends, including Groth16, PLONK, and Marlin, focusing on throughput metrics relevant to blockchain applications. Their study provided valuable cross-implementation comparisons but did not address the specific requirements of sensor network environments, such as energy constraints and intermittent connectivity.

The Circom ecosystem, which forms the basis of our experimental framework, has been evaluated in several contexts. Iden3 [18] released comprehensive documentation and benchmarks for the circomlib circuit library, establishing baseline performance expectations for standard cryptographic primitives. El-Hajj and Oude Roelink [19] conducted a comparative benchmark of zk-SNARK, zk-STARK, and Bulletproof protocols using a MiMC hash application, finding that zk-SNARK produced the smallest proofs while zk-STARK offered the fastest proof generation. Kuznetsov et al. [20] presented an earlier benchmarking analysis of Groth16 using the same Circom-snarkjs toolchain on seven circuit types, providing initial empirical data on proof lifecycle performance. In the domain of blockchain-based sensor networks, Kuznetsov et al. [21] proposed an OR-aggregation approach for zero-knowledge set membership proofs tailored to resource-constrained IoT environments. More recently, Jayaraman and Delhibabu [22] developed TeleZK-L2, a zk-SNARK framework for privacy-preserving telehealth data verification on the Layer-2 blockchain, demonstrating the growing relevance of ZKPs in healthcare IoT. However, systematic evaluation across diverse circuit categories with attention to sensor network deployment requirements has been lacking.

### 2.3. Blockchain-Based Sensor Data Management

Blockchain platforms for sensor data management have been proposed by multiple research groups. Reyna et al. [23] surveyed blockchain applications in IoT, identifying data integrity verification as a primary use case while noting the computational overhead as a key barrier to adoption. Dai et al. [24] proposed a hierarchical blockchain architecture for sensor networks that distributes computational load between edge and cloud layers, an approach that aligns with our identified deployment tiers.

Recent work by Kang et al. [25] explored the integration of zk-SNARKs with lightweight blockchain protocols for vehicular sensor networks, demonstrating practical data verification with sub-second verification times. Their results, while encouraging, were limited to a specific application domain and did not generalize across circuit complexity classes.

### 2.4. Positioning of This Work

Our work distinguishes itself from prior studies in three key aspects. First, we provide the first comprehensive benchmarking study that systematically covers the full spectrum of circuit complexities relevant to sensor network applications, from trivial arithmetic to complex cryptographic protocols. Second, we characterize all phases of the proof lifecycle rather than focusing solely on proof generation or verification, enabling holistic resource planning. Third, we explicitly map our findings to sensor network deployment scenarios, providing actionable guidelines rather than abstract performance metrics.

## 3. Background

### 3.1. Zero-Knowledge Proofs

A zero-knowledge proof system allows a prover P to convince a verifier V that a statement x∈L for some language L∈NP without revealing the witness *w* that certifies membership. Formally, a ZKP system must satisfy three properties: *completeness* (an honest prover can always convince an honest verifier), *soundness* (a dishonest prover cannot convince the verifier of a false statement except with negligible probability), and *zero-knowledge* (the verifier learns nothing beyond the validity of the statement) [8].

### 3.2. zk-SNARKs and Groth16

Zero-knowledge succinct non-interactive arguments of knowledge (zk-SNARKs) are a class of ZKP constructions characterized by constant-size proofs and constant-time verification. The Groth16 protocol [9] achieves the most compact proofs among pairing-based zk-SNARK constructions, consisting of three group elements on the BN128 elliptic curve.

The Groth16 workflow operates on Rank-1 Constraint Systems (R1CS), where a computation is expressed as a system of constraints of the form:

(1) $$a_{i}\;\cdot \;s\times b_{i}\;\cdot \;s=c_{i}\;\cdot \;s,\;i=1,\ldots ,m$$

where s is the assignment vector containing public inputs, private inputs (witness), and intermediate variables, and $a_{i},b_{i},c_{i}$ are coefficient vectors. The number of constraints *m* determines the circuit complexity and directly influences proof generation time.

The Groth16 protocol requires a circuit-specific trusted setup [26] that produces a structured reference string (SRS) consisting of a proving key pk and a verification key vk. Given a witness *w* satisfying the R1CS, the prover computes a proof π=(A,B,C) where $A,C\in G_{1}$ and $B\in G_{2}$ are elliptic curve points. Verification requires a constant number of pairing operations independent of the circuit size, yielding O(1) verification complexity.

### 3.3. Relevance to Sensor Networks

The properties of Groth16 align particularly well with sensor network constraints:

- **Constant proof size**: Groth16 proofs consist of three elliptic curve elements totaling approximately 800 bytes on BN128, regardless of circuit complexity. This is critical for sensor networks where communication bandwidth may be limited to kilobytes per transaction.
- **Constant verification time**: Verification requires a fixed number of pairing operations, enabling deployment on resource-constrained gateway nodes that must validate proofs from multiple sensors.
- **Non-interactivity**: Proofs can be generated offline by sensor devices and transmitted asynchronously, accommodating intermittent connectivity patterns common in wireless sensor networks.
- **Asymmetric computation**: The computational burden falls on the prover (sensor or edge device generating the proof) while verification is lightweight, enabling efficient hub-and-spoke architectures where a central verifier validates proofs from many sensors.

## 4. Methodology

### 4.1. Benchmarking Framework Architecture

We developed an open-source automated benchmarking framework that implements the complete Groth16 proof lifecycle and collects detailed performance metrics at each stage. The framework is built on the Circom circuit compiler (version 2.1.9) and the snarkjs proof system library (version 0.7.4), which together constitute the most widely used open-source zk-SNARK toolchain.

The framework architecture consists of five modular components:

1. **Circuit Compiler**: Translates Circom circuit descriptions into R1CS constraint systems and WebAssembly (WASM) witness generators, with dependency tracking and build caching for reproducible compilation.
2. **Setup Manager**: Handles the trusted setup ceremony, including Powers of Tau generation, phase-2 circuit-specific setup, and key management with automatic PTAU power selection based on circuit complexity.
3. **Prover**: Performs witness generation from circuit inputs via WASM execution, followed by Groth16 proof generation, with integrated memory profiling.
4. **Verifier**: Validates generated proofs against verification keys and public inputs, with precise timing measurements.
5. **Analysis Engine**: Processes benchmark results to generate statistical summaries, regression analyses, and visualization charts.

### 4.2. Circuit Selection and Classification

We selected eight circuit types organized into four categories that represent common computational patterns in sensor network verification scenarios (Table 1).

**Arithmetic circuits**(multiply, quadratic) serve as baselines for measuring framework overhead and represent the simplest verification tasks, such as proving correct multiplication of calibration factors or polynomial evaluation of sensor transfer functions.

**Range proof circuits** implement bit decomposition to prove that a value lies within a specified range without revealing the value. This is directly applicable to sensor data verification scenarios such as proving a temperature reading is within a safe operating range or a patient’s vital sign is within normal parameters.

**Cryptographic hash circuits** (Poseidon and MiMC) compute hash functions optimized for arithmetic circuits, enabling data integrity verification and commitment schemes. Poseidon [27] is specifically designed for zk-SNARK efficiency, while MiMC provides an alternative with different performance characteristics. These circuits underpin privacy-preserving data aggregation protocols.

**Structural proof circuits** implement Merkle tree membership proofs at two scales: depth-4 (16 leaves, 980 constraints) and depth-16 (65,536 leaves, 3890 constraints). Merkle proofs are fundamental to set membership verification, enabling applications such as proving a sensor belongs to an authorized device list or that a data record exists in a verifiable database.

**Advanced cryptographic circuits** represent the upper bound of computational complexity. The ECDSA signature verification circuit (1,510,185 constraints on secp256k1) demonstrates the feasibility of verifying standard digital signatures within a zero-knowledge proof, enabling anonymous authentication protocols.

### 4.3. Measurement Protocol

We employed a controlled experimental protocol that distinguishes between *cold* and *warm* execution phases. The cold phase (1 execution per circuit) measures first-run performance including full compilation and setup from scratch, capturing initialization overhead. The warm phase (20 iterations per circuit) reuses pre-compiled artifacts and cached setup parameters, isolating the core proof lifecycle performance that is representative of steady-state operation in deployed sensor systems.

For each warm iteration, the framework copies pre-built compilation artifacts (R1CS and WASM), cryptographic keys, and input files to a fresh working directory, ensuring measurement independence while avoiding redundant setup computation. All 160 warm-phase measurements constituted the primary dataset for statistical analysis.

Timing measurements used monotonic high-resolution timers (time.perf_counter()) with microsecond precision. The measured phases are:

- **Compilation time** ($t_{\mathrm{compile}}$): Circom source to R1CS and WASM artifacts.
- **Setup time** ($t_{\mathrm{setup}}$): Groth16 key generation from R1CS and PTAU.
- **Witness generation time** ($t_{\mathrm{witness}}$): Input processing through WASM execution.
- **Proof generation time** ($t_{\mathrm{prove}}$): Groth16 proof computation from witness and proving key.
- **Verification time** ($t_{\mathrm{verify}}$): Proof validation using the verification key.
- **Total time** ($t_{\mathrm{total}}$): End-to-end execution including all phases.

Additionally, we recorded: proof size in bytes (|π|), public input size (|x|), peak resident set size (RSS) memory consumption, and constraint count (*m*) from R1CS compilation. Statistical analysis includes 95% confidence intervals computed using the Student *t*-distribution, coefficients of variation (CV), one-way ANOVA with $\eta^{2}$ effect sizes, Shapiro–Wilk normality tests, and pairwise Welch *t*-tests with Cohen’s *d* effect sizes.

### 4.4. Experimental Environment

All benchmarks were executed on a desktop system running Windows with an x86_64 processor (multi-core), 16 GB RAM, and SSD storage. The software stack consisted of Circom compiler 2.1.9, snarkjs 0.7.4, Node.js v22.19.0, and Python 3.10+. Each circuit was measured with 1 cold-start execution followed by 20 warm-phase iterations, yielding 168 total measurements. All timing data include only the computational time of the respective phase, excluding I/O and framework overhead. The benchmarking framework, experiment orchestration script, and all circuit implementations are publicly available at https://github.com/KuznetsovKarazin/zksnark-benchmark (accessed on 15 April 2026).

## 5. Results

### 5.1. Overall Performance Characteristics

All 168 benchmark executions (8 cold + 160 warm) completed successfully with 100% proof verification rate, confirming the functional correctness and reliability of the experimental setup. Table 2 presents the complete warm-phase performance statistics across all eight circuits.

The data reveal several important patterns. Proof generation time ($t_{\mathrm{prove}}$) ranges from 0.686 s for range proofs (32 constraints) to 47.9 s for ECDSA verification (1,510,185 constraints), representing a 70× increase in proving time for a 47,193× increase in constraint count—a strongly sub-linear relationship. Verification time remains remarkably stable at a grand mean of 0.662±0.032 s (CV = 4.9%) across all 160 measurements, confirming the theoretical O(1) verification complexity of Groth16 [9]. Proof size is effectively constant at 804.7±1.7 bytes (CV = 0.21%).

### 5.2. Measurement Reproducibility

**Figure 1:**
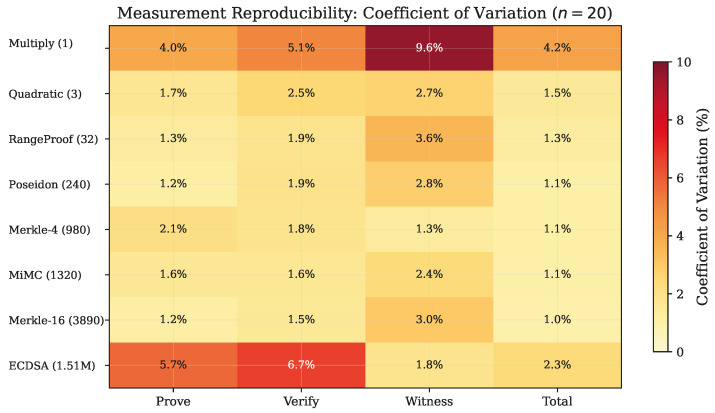
Coefficientof variation heatmap for all timing metrics across circuit types (*n* = 20). Lower values (lighter) indicate higher measurement reproducibility.

The controlled warm-phase protocol yields highly reproducible measurements. Figure 1 presents the coefficient of variation (CV) across all metrics and circuits as a heatmap.

For six of the seven non-ECDSA circuits, proof generation CV ranges from 1.2% to 2.1%, indicating excellent reproducibility. The multiply circuit exhibits slightly higher variability (CV = 4.0%) likely due to its minimal computation being more susceptible to system-level noise. The ECDSA circuit shows CV = 5.7% for prove time, consistent with the longer execution exposing more operating system scheduling variability. Shapiro–Wilk normality tests confirm that prove time distributions are consistent with normality for all circuits except ECDSA (W=0.727, p=0.0001), which exhibits a right-skewed distribution.

### 5.3. Scaling Analysis

**Figure 2:**
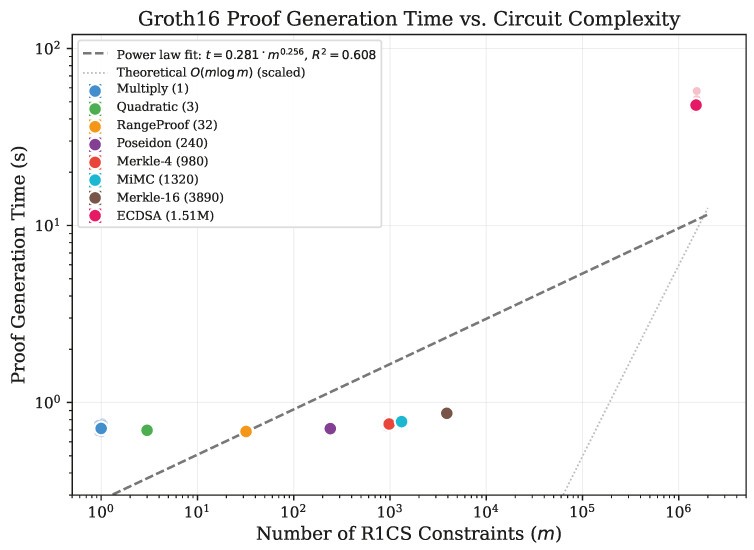
Proof generation time versus circuit complexity (log–log scale). Individual data points (*n* = 20 per circuit) shown with jitter; solid markers indicate means with 95% CI error bars. Dashed line: power law fit; dotted line: theoretical *O*(*m*log*m*) (scaled).

To quantify the relationship between circuit complexity and computational cost, we performed log–log regression analysis on the mean warm-phase timing data. We modeled the relationship as a power law: $t=k\;\cdot \;m^{\alpha }$, where *t* is execution time, *m* is constraint count, *k* is a constant factor, and α is the scaling exponent. Figure 2 presents the scaling analysis with individual data points.

The regression yields α=0.256 (k=0.282, $R^{2}=0.608$) for proof generation and α=0.317 (k=0.473, $R^{2}=0.608$) for total execution time. These scaling exponents represent an empirical fit within the specific Circom/snarkjs implementation and hardware configuration used in this study, rather than a general scaling law for Groth16 performance across all implementations and sensor network workloads. The moderate $R^{2}$ reflects the bimodal structure of the data: circuits up to 3890 constraints cluster in a narrow time band (0.69–0.87 s), while the ECDSA circuit at 1.5M constraints lies far above. Spearman rank correlation confirms a strong monotonic relationship (ρ=0.833, p=0.010).

### 5.4. Statistical Significance of Circuit Category Effects

One-way ANOVA on proof generation times across the seven non-ECDSA circuits reveals highly significant differences (F=355.0, $p<10^{-79}$), with circuit type explaining 94.1% of variance ($\eta^{2}=0.941$). This indicates that circuit structure, rather than random variation, is the dominant factor determining proof generation performance.

Pairwise Welch *t*-tests with Cohen’s *d* effect sizes quantify the practical significance of differences between selected circuit pairs (Table 3).

**Figure 3:**
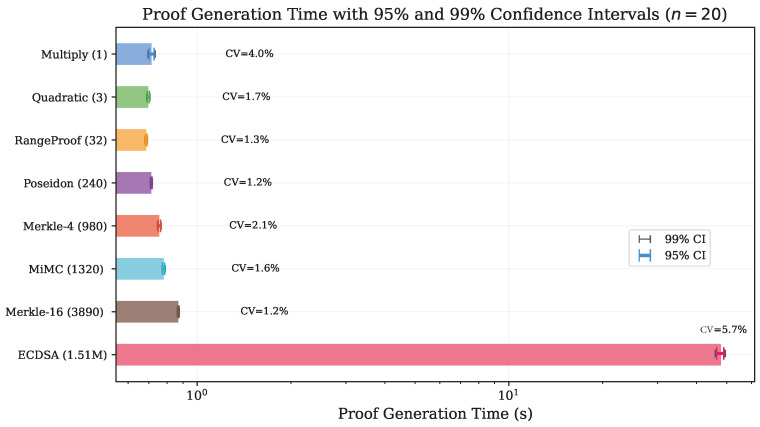
Mean proof generation time with 95% (thick bar) and 99% (thin bar) confidence intervals for all eight circuits (*n* = 20). CV annotations confirm that measurement variability remains below 6% across all circuits, validating the experimental methodology.

The very large effect sizes (|d|>2) for most pairs confirm that even modest time differences between circuits are highly significant given the low measurement variability. Figure 3 summarizes the statistical precision achieved for each circuit with 95% and 99% confidence intervals.

### 5.5. Verification Time Stability

**Figure 4:**
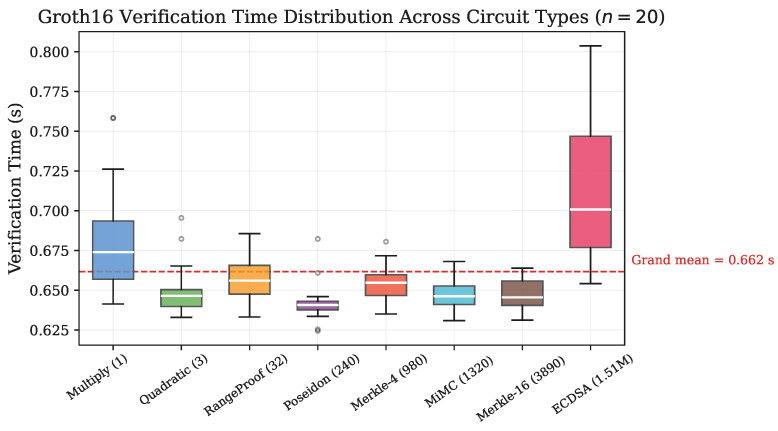
Box plot of verification time distributions across all circuit types (*n* = 20). The dashed red line indicates the grand mean (0.662 s). Despite spanning six orders of magnitude in constraint count, verification time remains within the 0.62–0.80 s range.

Figure 4 presents the distribution of verification times across all eight circuits, demonstrating the constant-time verification property of Groth16.

While ANOVA detects statistically significant differences in verification time across circuits (F=21.82, $p<10^{-19}$, $\eta^{2}=0.50$), the practical magnitude of these differences is negligible: the range of circuit-level means spans only 72 ms (from 0.642 s for Poseidon to 0.714 s for ECDSA). This residual variation likely reflects minor differences in public input processing overhead rather than circuit-dependent verification complexity.

### 5.6. Proof Size Constancy

**Figure 5:**
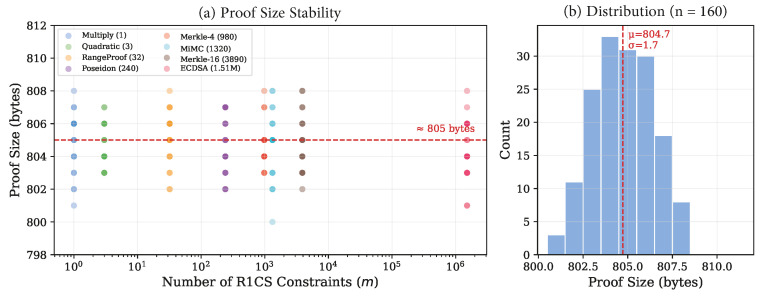
(**a**) Proof size versus constraint count for all 160 warm-phase measurements. (**b**) Histogram of proof sizes across all circuits, confirming the near-constant size property (*μ* = 804.7, *σ* = 1.7 bytes).

Figure 5 confirms the constant proof size property with 160 individual measurements. All proofs fall within the 798–812 byte range, with a mean of 804.7 bytes and standard deviation of 1.7 bytes. The minor variations are attributable to JSON serialization formatting of the three elliptic curve elements ($\pi_{A}\in G_{1}$, $\pi_{B}\in G_{2}$, $\pi_{C}\in G_{1}$) and differences in the magnitude of serialized field element digits.

### 5.7. Phase-Wise Time Decomposition

**Figure 6:**
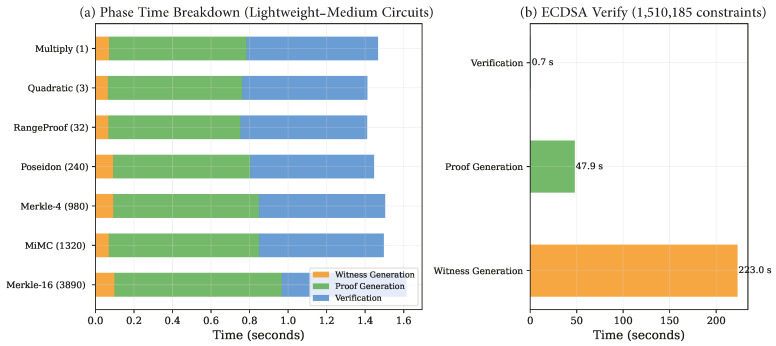
(**a**) Stacked time breakdown for non-ECDSA circuits. (**b**) Phase decomposition for the ECDSA circuit, where witness generation (223.0 s) dominates due to WebAssembly evaluation of 1.5 M constraints.

Figure 6 decomposes total execution time into its constituent phases. For the seven non-ECDSA circuits, proof generation (42–54%) and verification (39–48%) dominate execution time in roughly equal proportions, with witness generation contributing 4–7%.

The ECDSA circuit presents a qualitatively different profile: witness generation (223.0 s, 82.1%) dominates, followed by proof generation (47.9 s, 17.6%), with verification contributing only 0.3% of total time. This phase decomposition is critical for deployment planning, as it reveals that the bottleneck for complex circuits lies in witness computation rather than the cryptographic proving algorithm itself.

### 5.8. Proof Generation Efficiency

**Figure 7:**
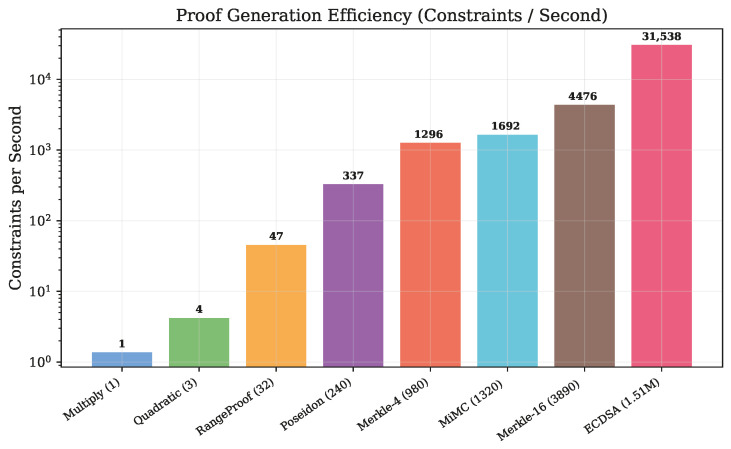
Proof generation efficiency (constraints per second) by circuit type. Efficiency scales super-linearly with circuit complexity, from 1.4 constraints/s for multiply to 31,535 constraints/s for ECDSA, indicating that the Groth16 prover achieves better amortization for larger circuits.

Figure 7 presents the proof generation efficiency metric (constraints processed per second), revealing a strong positive relationship with circuit complexity.

The efficiency ranges from 1.4 constraints/s (multiply) to 31,535 constraints/s (ECDSA), a 22,500× improvement. This super-linear efficiency gain arises because the constant overhead of the JavaScript runtime and proof serialization is amortized across more constraints in larger circuits.

### 5.9. Prove Time Distributions

**Figure 8:**
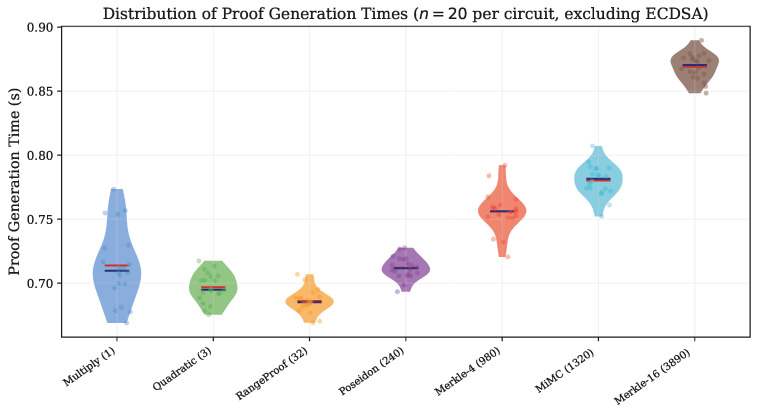
Violin plots with overlaid individual measurements for proof generation time across non-ECDSA circuits (*n* = 20 each). Red markers: mean; blue markers: median. The progressive widening from range_proof to merkle_poseidon illustrates the gradual increase in mean prove time with circuit complexity.

Figure 8 presents violin plots of proof generation time distributions for the seven non-ECDSA circuits, combining density estimation with individual data points.

## 6. Discussion

### 6.1. Proposed Architecture for Blockchain-Anchored Sensor Data Verification

**Figure 9:**
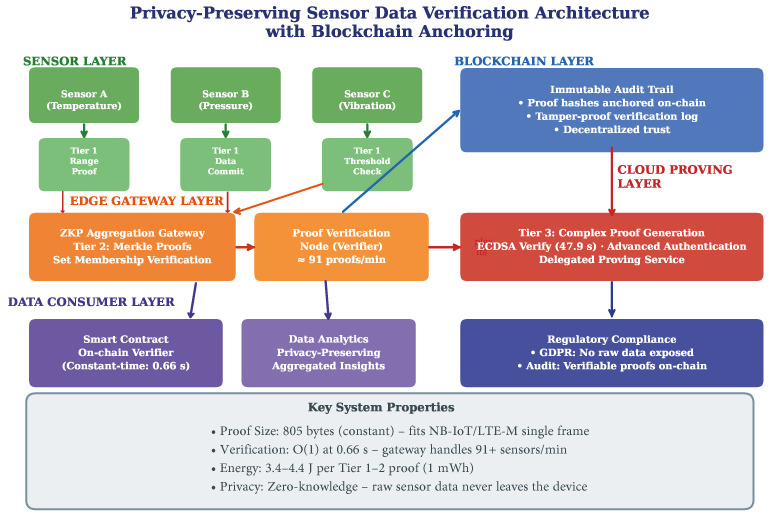
Proposed five-layer architecture for privacy-preserving sensor data verification with blockchain anchoring. The architecture maps circuit complexity tiers to infrastructure layers, with constant-size proofs (∼805 bytes) enabling efficient cross-layer communication.

Beyond benchmarking, our results motivate a concrete reference architecture for privacy-preserving sensor data verification with blockchain integration. We emphasize that this architecture is a conceptual proposal informed by our desktop-based empirical measurements; its layers and tier assignments have not been validated on actual IoT hardware or in a deployed sensor network. Figure 9 presents a five-layer architecture that leverages the empirical performance characteristics established in Section 5.

The **Sensor Layer** hosts resource-constrained devices that generate Tier 1 zk-SNARK proofs locally. Each sensor proves statements about its data (e.g., “temperature is within safe range”) without transmitting raw readings. Our measurements confirm that Tier 1 proofs require under 0.72 s and approximately 3.5 J on our desktop platform, suggesting that on-device proving may be feasible for mains-powered or periodically recharged sensors, although performance on ARM-based IoT hardware requires direct validation.

The **Edge Gateway Layer** aggregates proofs from multiple sensors and performs two functions: (1) verification of incoming Tier 1 proofs at the measured rate of 91 proofs per minute and (2) generation of Tier 2 aggregation proofs using Merkle tree circuits. The Merkle membership proof (3890 constraints, 0.87 s prove time) enables efficient set membership verification—a gateway can prove that all contributing sensors belong to an authorized device registry without revealing individual identities.

The **Blockchain Layer** receives proof hashes from the gateway and anchors them in an immutable ledger, providing a tamper-proof audit trail. The architecture naturally supports smart contract-based verification [28]. Critically, only proof hashes (32 bytes) and verification results are stored on-chain, not the proofs themselves (805 bytes) or the raw data. This design minimizes blockchain storage costs while preserving verifiability: any auditor can retrieve the proof from off-chain storage, recompute its hash, and verify it matches the on-chain record. The constant-time Groth16 verification (0.66 s) enables efficient on-chain verification through smart contracts.

The **Cloud Proving Layer** handles Tier 3 computations that exceed edge device capabilities, such as ECDSA signature verification for anonymous device authentication. Our results show that while Tier 3 proving is computationally expensive (47.9 s), verification remains constant-time, enabling a delegated proving model where cloud infrastructure generates proofs that edge devices can efficiently validate.

The **Data Consumer Layer** encompasses applications that consume verified, privacy-preserving data: smart contract verifiers, analytics platforms operating on aggregated (not individual) data, and regulatory compliance systems. The architecture is designed to support GDPR compliance [29] by ensuring that raw sensor data never leaves the originating device—only zero-knowledge proofs traverse the network.

This architecture’s key innovation is the systematic mapping of zk-SNARK deployment tiers to network infrastructure layers, informed by our empirical benchmarking data. The constant proof size (805 bytes) serves as a universal communication primitive across all layers, while the constant verification time (0.66 s) enables decentralized verification at any layer.

### 6.2. Illustrative Use Case: Smart Building Environmental Monitoring

To concretize the proposed architecture, we describe an end-to-end scenario for blockchain-anchored environmental monitoring in a smart building with privacy-preserving compliance reporting.

**Scenario.** A commercial building deploys 200 temperature, humidity, and CO_2_ sensors across 50 zones. Regulatory compliance (e.g., workplace safety directives) requires proving that environmental conditions remain within prescribed limits, while building operators wish to keep precise readings confidential (competitive or tenant-privacy reasons).

**Step 1: On-device proof generation (Tier 1).** Each sensor periodically generates a range proof (32 constraints, $t_{\mathrm{prove}}=0.686$ s) attesting that its reading falls within the regulatory threshold—e.g., proving 18≤T≤26 °C without revealing the exact temperature. The proof payload (805 bytes) is transmitted via the building’s LoRaWAN or Wi-Fi infrastructure alongside a Poseidon hash commitment to the raw value.

**Step 2: Gateway aggregation (Tier 2).** A floor-level edge gateway collects proofs from its zone’s sensors, verifies each proof (0.66 s per proof, all 200 sensors processable in 2.2 min), and generates a Merkle membership proof (3890 constraints, 0.87 s) attesting that all contributing sensors are from the authorized device registry. The gateway also computes an aggregated compliance attestation over the verified zone data.

**Step 3: Blockchain anchoring.** The gateway submits to a blockchain smart contract: (a) the Merkle root of verified proofs, (b) the aggregated compliance result, and (c) a hash linking to off-chain proof storage. The on-chain transaction requires only ∼100 bytes. A smart contract verifier confirms the Groth16 proof of the aggregation step in constant time (0.66 s), recording an immutable compliance record.

**Step 4: Audit and compliance.** A regulatory auditor queries the blockchain for the building’s compliance history. For any flagged period, the auditor retrieves the relevant proofs from off-chain storage, independently verifies them against the on-chain root, and confirms compliance—all without accessing a single raw sensor reading. GDPR requirements are inherently satisfied: no personal or operationally sensitive data leaves the building.

This workflow requires only Tier 1 and Tier 2 circuits, with total per-cycle energy cost under 5 J per sensor and blockchain storage under 200 bytes per reporting cycle. The entire proof pipeline from sensor reading to blockchain anchoring completes within the typical 15 min LoRaWAN duty cycle, confirming the practical viability demonstrated by our benchmarks.

### 6.3. Deployment Tiers for Sensor Networks

**Figure 10:**
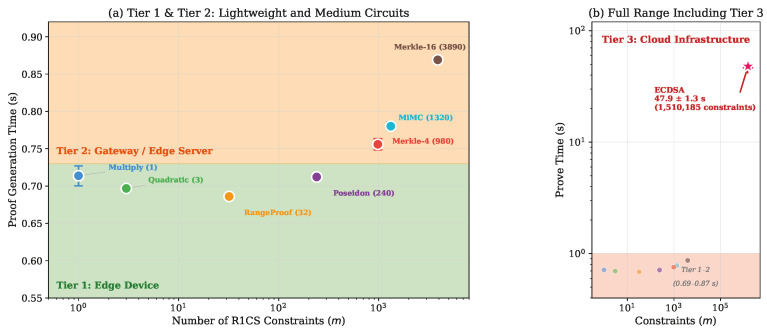
Sensor network deployment tiers. (**a**) Detailed view of Tier 1 (edge device) and Tier 2 (gateway) circuits showing proof generation times with 95% CI. (**b**) Full complexity range including Tier 3 (cloud), illustrating the scale separation between lightweight circuits and ECDSA verification. The star marker (⋆) in panel (**b**) denotes the ECDSA circuit (1,510,185 constraints; 47.9 s prove time), representing the sole Tier 3 data point.

Based on our benchmarking results, we identify three operational deployment tiers that map circuit complexity to appropriate computational infrastructure in sensor network architectures (Table 4). These tiers and their hardware mappings represent preliminary heuristic guidance derived from our desktop measurements; they should be interpreted as indicative rather than as experimentally validated deployment rules. Direct benchmarking on the target device classes is needed to confirm the estimated performance ranges. Figure 10 visualizes these tiers on the prove time versus constraint count plane.

**Tier 1 (Lightweight)** encompasses circuits with fewer than 100 constraints, including basic arithmetic validation and range proofs. These circuits achieve sub-second proof generation with low variability (CV ≤ 4%) and represent the most immediately deployable class for sensor-side proving. Practical applications include proving that sensor readings fall within acceptable calibration ranges and generating data commitments.

**Tier 2 (Medium)** covers circuits in the 100–5000 constraint range, including cryptographic hash computations and Merkle tree membership proofs. The prove time increase from Tier 1 to Tier 2 is statistically significant (Welch *t*: $p<10^{-10}$) but modest in absolute terms (under 200 ms additional). These circuits remain within the capabilities of gateway-class devices [30].

**Tier 3 (Heavy)** addresses circuits exceeding 5000 constraints, exemplified by ECDSA signature verification. These circuits require substantial computational resources (47.9 s mean prove time for ECDSA) and are best suited for cloud-based proving servers. Despite the high proving cost, the constant verification time (0.71 s) means that proof validation can still be performed at the edge.

**Figure 11:**
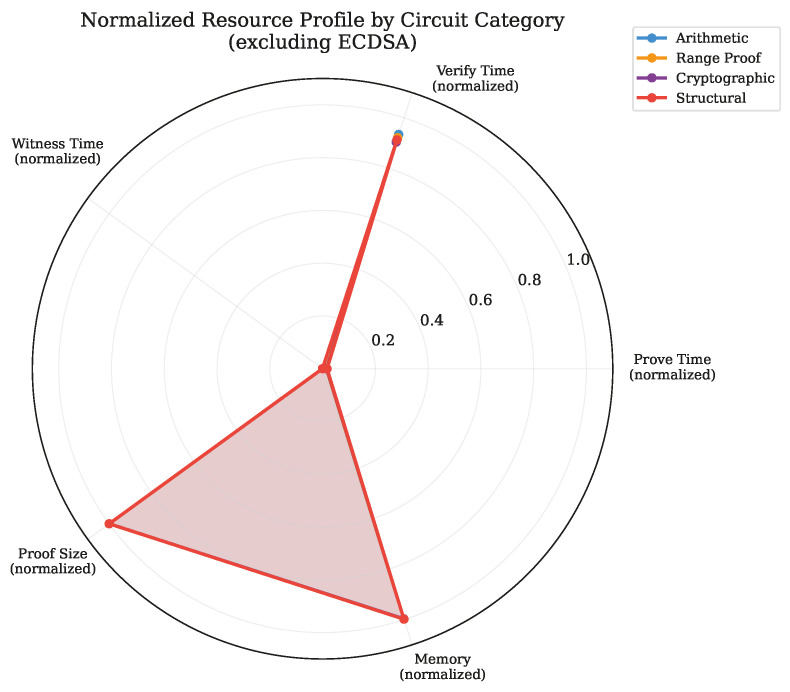
Normalized resource consumption profiles for Tier 1–2 circuit categories across five dimensions. All values are normalized to the maximum observed across categories. The compact profiles of arithmetic and range proof categories confirm their suitability for resource-constrained IoT sensor devices.

Figure 11 presents normalized resource profiles for each circuit category, enabling visual comparison of the computational footprint across the five key dimensions relevant to IoT deployment planning.

### 6.4. Energy Budget Analysis

For battery-powered sensor deployments, energy consumption is a critical constraint. Table 5 presents estimated energy budgets for proof generation assuming a 5 W computational power draw, representative of ARM Cortex-A class edge processors during cryptographic workloads.

For Tier 1 and Tier 2 circuits, the energy cost per proof (3.4–4.4 J) is comparable to a single LoRaWAN transmission (approximately 0.5–1.5 J at typical power settings). This suggests that incorporating zk-SNARK proving into sensor duty cycles could be energetically feasible for mains-powered or frequently recharged devices, pending validation on target hardware. A sensor with a 3000 mAh battery at 3.7 V (approximately 40 kJ capacity) could generate over 9000 Tier 1 proofs before battery depletion, assuming proof generation constitutes the sole energy expenditure.

### 6.5. Compatibility with IoT Communication Protocols

A key practical consideration for deploying zk-SNARKs in sensor networks is compatibility with existing IoT communication protocols. Table 6 maps the measured proof characteristics to the payload constraints of widely deployed IoT protocols.

The constant proof size of ∼805 bytes (JSON) or ∼256 bytes (binary-encoded three BN128 curve elements) is directly compatible with cellular IoT (NB-IoT [32], LTE-M) and Wi-Fi without any adaptation. For LPWAN protocols with tighter payload and security constraints [33], binary proof serialization reduces the payload to approximately 256 bytes—within a single LoRaWAN frame at SF7. Application-layer protocols such as MQTT [34] and CoAP can carry proofs as structured payloads with standard QoS mechanisms.

For the verification side, the constant O(1) verification time of 0.66 s is compatible with typical IoT duty cycles. A LoRaWAN Class A device transmitting every 15 min generates 96 proofs per day; a single gateway verifier can handle the proof stream from over 1300 such devices at the measured verification rate of 91 proofs per minute.

### 6.6. IoT Device Class Mapping

Table 7 maps the identified deployment tiers to representative IoT hardware classes, providing concrete guidance for practitioners.

### 6.7. Comparison with Alternative Approaches

Our benchmarking results allow qualitative comparison with alternative privacy-preserving verification approaches. Traditional digital signatures (ECDSA and EdDSA) provide data authenticity in 64–72 bytes with sub-millisecond verification but no privacy. Homomorphic encryption enables computation on encrypted data but incurs orders-of-magnitude higher computational and communication overhead. Trusted execution environments (TEEs) provide hardware-based privacy with minimal computational overhead but require specific hardware support. zk-SNARKs occupy a unique position offering mathematical privacy guarantees with moderate computational cost and no hardware dependencies.

### 6.8. Limitations

Several limitations should be acknowledged. First, all benchmarks were conducted on desktop-class Windows hardware (x86_64 multi-core processor, 16 GB RAM, SSD storage), which differs significantly from typical sensor network environments; performance on ARM-based IoT devices may differ significantly, and consequently the deployment tier assignments and energy estimates presented in this work should be treated as preliminary guidance rather than validated deployment specifications. Second, we evaluated only Groth16; newer constructions (PLONK [36], Halo2, zk-STARKs) may offer different trade-offs. Third, the ECDSA circuit exhibits non-normal prove time distributions (W=0.727, p<0.001), suggesting that parametric confidence intervals should be interpreted cautiously for this circuit. Fourth, energy estimates are time-based approximations; direct power measurements on target hardware would improve accuracy. Fifth, our proof sizes reflect JSON serialization; binary encoding would reduce proof payload to approximately 256 bytes.

## 7. Conclusions

This paper presented a systematic empirical evaluation of the Groth16 zk-SNARK protocol across eight representative circuit types relevant to sensor network data verification. Our benchmarking study, conducted using an automated open-source framework with 160 statistically controlled measurements (20 warm-phase iterations per circuit), provides comprehensive performance data spanning six orders of magnitude in circuit complexity.

The key findings are as follows. First, Groth16 proofs maintain a constant size of 804.7±1.7 bytes (CV = 0.21%) regardless of circuit complexity, confirming their suitability for bandwidth-constrained sensor communication channels. Second, verification time is effectively constant at 0.662±0.032 s (CV = 4.9%) across all circuit types, enabling scalable proof validation on resource-constrained verifier devices at rates exceeding 90 proofs per minute. Third, proof generation time exhibits favorable sub-linear scaling (α=0.256), with proving times ranging from 0.686 s for range proofs to 47.9 s for ECDSA signature verification and highly significant circuit-dependent differences (F=355.0, $\eta^{2}=0.94$). Fourth, measurement reproducibility is excellent, with coefficients of variation below 2.1% for proof generation across six of the eight circuits, validating our experimental methodology for future comparative studies.

These results suggest that zk-SNARKs represent a promising technology for privacy-preserving data verification in sensor networks, although validation on representative IoT hardware is required to confirm deployment feasibility. Lightweight verification tasks (range proofs and data commitments) require only 3.4–3.6 J per proof at estimated 5 W computational draw, which may make them feasible for mains-powered edge devices. The three deployment tiers identified provide actionable guidance for matching verification complexity to available computational infrastructure. Furthermore, the proposed five-layer architecture demonstrates how these empirical findings translate into a practical system design with blockchain anchoring for immutable audit trails and GDPR-compliant data processing.

Future work will extend this study in several directions: (1) performance evaluation on representative IoT hardware platforms (ARM Cortex-M and Cortex-A); (2) comparative analysis with PLONK, Halo2, and zk-STARKs; (3) direct energy consumption profiling on battery-powered devices; (4) binary proof serialization to reduce payload to ∼256 bytes for LPWAN compatibility; (5) development of optimized circuit templates for common sensor data verification patterns; and (6) prototype implementation of the proposed blockchain-anchored architecture on a physical IoT testbed with end-to-end latency and throughput characterization.

## Figures and Tables

**Table 1:** Selected benchmark circuits and their characteristics. Constraint counts are determined by R1CS compilation.

Category	Circuit	Constraints	PTAU Power	Sensor Network Use Case
Arithmetic	multiply	1	8	Baseline overhead measurement
	quadratic	3	8	Polynomial data validation
Range Proof	range_proof	32	8	Threshold verification ($0\leq x<2^{32}$)
Cryptographic	poseidon_hash	240	8	Data integrity commitment
	hash (MiMC)	1320	11	Chained hash verification
Structural	merkle_poseidon_4	980	10	Set membership (16 leaves)
	merkle_poseidon	3890	12	Set membership (65,536 leaves)
Advanced	ecdsa_verify	1,510,185	21	Digital signature authentication

**Table 2:** Warm-phase benchmark results (*n* = 20 per circuit). Times in seconds (mean ± std); proof size in bytes; memory in MB. CI denotes the 95% confidence interval half-width.

Circuit	*m*	*t*_prove_ (Mean ± Std)	CI_95_	*t*_verify_ (Mean ± Std)	*t*_witness_	|π|	RSS
multiply	1	0.714 ± 0.029	0.013	0.683 ± 0.035	0.071	805	23.7
quadratic	3	0.697 ± 0.012	0.005	0.650 ± 0.016	0.065	805	23.6
range_proof	32	0.686 ± 0.009	0.004	0.657 ± 0.012	0.067	805	23.7
poseidon_hash	240	0.712 ± 0.009	0.004	0.642 ± 0.012	0.092	805	23.6
merkle_poseidon_4	980	0.756 ± 0.016	0.007	0.655 ± 0.012	0.093	805	23.7
hash (MiMC)	1320	0.780 ± 0.012	0.006	0.648 ± 0.010	0.069	805	23.7
merkle_poseidon	3890	0.869 ± 0.010	0.005	0.647 ± 0.010	0.098	805	23.7
ecdsa_verify	1,510,185	47.884 ± 2.731	1.278	0.714 ± 0.048	222.992	804	23.7

**Table 3:** Pairwise Welch *t*-test results for proof generation time between selected circuit pairs.

Circuit A	Circuit B	Δ$\bar{t}$ (ms)	*t*	*p*	Cohen’s *d*
multiply	quadratic	16.9	2.45	0.022	0.78
range_proof	poseidon_hash	−26.1	−9.33	<10^−10^	−2.95
poseidon_hash	hash	−68.1	−20.20	<10^−19^	−6.39
hash	merkle_poseidon	−88.9	−24.83	<10^−23^	−7.85

**Table 4:** Proposed deployment tiers for zk-SNARK-based sensor data verification.

Tier	Constraints	*t*_prove_ (s)	Infrastructure	Applications
Lightweight	<100	<0.72	Edge/sensor device	Range proofs, threshold checks
Medium	100–5000	0.71–0.87	Gateway/edge server	Hash verification, Merkle proofs
Heavy	>5000	>1	Cloud/off-chain	Signature verification, complex logic

**Table 5:** Estimated energy consumption for proof generation per circuit type (assuming 5 W computational draw).

Circuit	*m*	*t*_prove_ (s)	Energy (J)	Energy (mWh)
range_proof	32	0.686	3.43	0.95
poseidon_hash	240	0.712	3.56	0.99
merkle_poseidon_4	980	0.756	3.78	1.05
merkle_poseidon	3890	0.869	4.35	1.21
ecdsa_verify	1,510,185	47.884	239.42	66.51

**Table 6:** Compatibility of Groth16 proof payload (∼805 bytes JSON, ∼256 bytes binary) with IoT communication protocols.

Protocol	Max Payload	JSON Proof	Binary Proof	Deployment Note
IEEE Std 802.15.4-2020 [31] (Zigbee)	127 B	Fragmented (7×)	Fragmented (2×)	Requires application-layer fragmentation
LoRaWAN (SF7/BW125)	242 B	Fragmented (4×)	Single frame	Binary serialization recommended
Bluetooth LE (5.x)	251 B	Fragmented (4×)	Single frame	Suitable for wearable sensors
NB-IoT	1600 B	Single frame	Single frame	Directly compatible
LTE-M	1600 B	Single frame	Single frame	Directly compatible
Wi-Fi (TCP/UDP)	1460+ B	Single frame	Single frame	Directly compatible
MQTT (over TCP)	256 MB	Single message	Single message	Directly compatible; QoS 1 recommended
CoAP (over UDP)	1024 B	Single block	Single block	Block-wise transfer for JSON

**Table 7:** Mapping of zk-SNARK deployment tiers to representative IoT device classes.

Tier	Device Class	Representative Hardware	Est. Prove Time	Suitable Circuits
1	MCU with crypto	ESP32-S3, nRF5340	2–5 s *	Range proof, threshold check
1	Single-board computer	Raspberry Pi Zero 2W	1–3 s *	Range proof, polynomial validation
2	Edge gateway	Raspberry Pi 4/5, Jetson Nano	1–2 s *	Poseidon hash, Merkle-4
2	Industrial gateway	Intel NUC, ARM Cortex-A72	0.8–1.5 s *	Merkle-16, MiMC hash chain
3	Cloud/edge server	x86 server, GPU-accelerated	30–60 s	ECDSA, complex authentication

* Estimated based on typical JavaScript runtime performance scaling from our desktop measurements; native implementations (e.g., rapidsnark [35]) may achieve 3–10× speedup.

## Data Availability

The benchmarking framework, circuit implementations, and raw benchmark data are publicly available at https://github.com/KuznetsovKarazin/zksnark-benchmark (accessed on 15 April 2026).

## Abbreviations

The following abbreviations are used in this manuscript:

ANOVA	Analysis of Variance
BLE	Bluetooth Low Energy
CoAP	Constrained Application Protocol
ECDSA	Elliptic Curve Digital Signature Algorithm
GDPR	General Data Protection Regulation
IoT	Internet of Things
LPWAN	Low-Power Wide-Area Network
MQTT	Message Queuing Telemetry Transport
NB-IoT	Narrowband Internet of Things
R1CS	Rank-1 Constraint System
RSS	Resident Set Size
SRS	Structured Reference String
WASM	WebAssembly
ZKP	Zero-Knowledge Proof
zk-SNARK	Zero-Knowledge Succinct Non-Interactive Argument of Knowledge
